# Dissociated Response to Immune Checkpoint Inhibition in Head and Neck Squamous Cell Carcinoma Is Common and Exhibits Distinct Clinical Outcomes

**DOI:** 10.1158/2767-9764.CRC-26-0363

**Published:** 2026-10-08

**Authors:** Alice N. Weaver, Stephen B. Keysar, Aarti Dureja, Tugs-Saikhan Chimed, Phuong N. Le, Cera Nieto, Nathaniel Alzofon, J. Jason Morton, Carissa M. Thomas, Ryan M. Lanning, Von G. Samedi, Adrie Van Bokhoven, Antonio Jimeno

**Affiliations:** 1 Division of Medical Oncology, Department of Medicine, University of Colorado Denver School of Medicine (UCDSOM), Aurora, Colorado.; 2 University of Colorado Cancer Center, UCDSOM, Aurora, Colorado.; 3 Department of Otolaryngology, UCDSOM, Aurora, Colorado.; 4 Department of Radiation Oncology, UCDSOM, Aurora, Colorado.; 5 Department of Pathology, UCDSOM, Aurora, Colorado.; 6 Gates Center for Regenerative Medicine, UCDSOM, Aurora, Colorado.

## Abstract

Immune checkpoint inhibition (ICI) constitutes the first-line therapy for relapsed/metastatic (R/M) head and neck squamous cell carcinoma (HNSCC) despite low response rates. Some patients treated with ICI experience dissociated response (DR), the coexistence of responding and progression lesions. DR prevalence and prognostic impact in HNSCC are unknown. We retrospectively identified 112 patients treated with ICI at our institution and evaluated their disease characteristics, survival outcomes, and response. Tissue specimens were used to assess the tumor microenvironment (TME). DR occurred in 19% of our patient cohort. DR was more likely in patients with disease involving the cervical lymph nodes [odds ratio (OR), 16.79; *P* = 0.004]. Patients with DR had a median progression-free and overall survival of 6.7 and 16.9 months, respectively, similar to subjects with partial response (PR) or stable disease (SD). DR was less favorable when it occurred early, and we identified no instances in which early DR converted to uniform objective response. We demonstrated increased CD8^+^ T-cell infiltration and decreased signal transducer and activator of transcription 3 (STAT3) signaling in tissue from responding versus nonresponding patients. Tumors from patients with DR had low T-cell infiltration and reduced STAT3. In conclusion, DR during ICI therapy in HNSCC is common and associated with favorable prognosis, especially in patients who initially have a complete response (CR) or PR. Early DR as the best response is less beneficial and unlikely to convert to a true response; change in therapy should be considered for these patients. Response patterns may be related to organ-specific differences in the TME.

**Significance:**

Mixed response or DR is common in patients with R/M HNSCC who receive immune checkpoint inhibition (ICI). Patients with DR have survival outcomes similar to patients with a PR, especially when DR occurs after an initial uniform response to therapy. DR occurring early in treatment is less favorable and unlikely to convert to uniform response later on.

## Introduction

Head and neck squamous cell carcinoma (HNSCC) accounts for 750,000 new cases worldwide each year (1). Locoregional disease is often curable with surgical resection, radiation, and/or chemotherapy. However, recurrent or metastatic (R/M) HNSCC is still a major cause of mortality. KEYNOTE-048 established immune checkpoint inhibition (ICI) with pembrolizumab, with or without doublet chemotherapy, as the standard of care in the first line (2). Unfortunately, the response rate for maximum intensity treatment with pembrolizumab plus chemotherapy was only 36%, with a median overall survival (OS) of about 13 months (2). Patients who have a low combined positive score (CPS) and/or are not candidates for chemotherapy have even lower likelihood of response (3). There are limited therapeutic options after ICI progression.

One challenge in the delivery of ICI is accurate assessment of response. Shortly after ICI introduction, clinicians noticed a phenomenon subsequently named “pseudoprogression,” characterized by an initial increase in size of some or all lesions followed by subsequent reduction in tumor burden. These changes are thought to be the result of localized inflammation, edema, hemorrhage, and necrosis (4). Depending on the cancer type, pseudoprogression may occur in up to 10% of patients (4), triggering a modified Response Evaluation Criteria in Solid Tumors (RECIST) for immune-based therapeutics (iRECIST) to standardize response assessments and prevent premature discontinuation of ICI.

A second atypical ICI response pattern has been recently identified in non–small cell lung cancer (NSCLC), referred to here as dissociated response (DR) but also known as heterogeneous or mixed response. This is the coexistence of responding and progressing lesions at the same imaging timepoint. DR can occur as part of an overall response of partial response (PR), stable disease (SD), or progressive disease (PD). The pattern can be seen in any cancer but may be more common in those with heterogeneous molecular profiles between metastatic deposits, such as NSCLC in which incidences between 10% and 30% are reported after both immunotherapy and epidermal growth factor receptor (EGFR) tyrosine kinase inhibitor therapy (5–10). The prevalence and prognostic significance of this finding are still unclear. ICI-related DR in NSCLC has typically been reported as favorable when compared with true/uniform disease progression, and most patients continued ICI past DR if symptoms were controlled with local therapies (7–9).

A hypothesis for DR is heterogeneity in the tumor microenvironment (TME), specifically with respect to immune phenotype. For example, Sobottka and colleagues (11) examined intrapatient matched primary breast tumors and distant metastases and found that a “hot” immune phenotype, defined by CD8^+^ T cells in direct contact with tumor cells, was more common in soft-tissue and brain metastases. In contrast, bone and liver metastases were more likely to have a “cold” immune phenotype. Decreased prevalence of CD8^+^ tumor-infiltrating T cells in bone metastases has been confirmed in other cancer types and seems to correspond clinically with lower bone disease ICI response rates (12–14). There has been no characterization of DR in HNSCC, but given the clinical heterogeneity, propensity for locoregional relapse, and increasing availability of effective local therapies such as hypofractionated reirradiation (15), it is critical to determine DR incidence and biological basis in HNSCC.

In this study, we describe patterns of ICI response in patients with R/M HNSCC. We identified a subgroup of patients with DR and reviewed the demographics, clinical factors, and outcomes associated with this finding. Finally, we profiled and compared the TME in primary, recurrent, and metastatic tumor biopsies from various organ sites to determine characteristics which may influence differential ICI response.

## Materials and Methods

### Patient identification, imaging evaluation, and tissue samples

Health Data Compass, a multi-institutional data warehouse, was used to retrospectively identify patients with HNSCC treated with ICI in the R/M setting at the University of Colorado Health Network from 2010 to 2022. Criteria included (i) at least three cycles of pembrolizumab, nivolumab, or durvalumab given as monotherapy or combined with chemotherapy, as the standard of care or a clinical trial, with or without palliative radiotherapy (RT); (ii) imaging evaluation (CT, MRI, or PET-CT) within 45 days prior to and 30 days after completion of three ICI cycles; and (iii) measurable disease. Data and tissue were collected under protocol #09-0179, reviewed and approved by the Colorado Multiple Institutional Review Board and conducted in accordance with recognized ethical guidelines. Tissue samples were archived diagnostic or surgical specimens. Tissue block adequacy was confirmed by a clinical pathologist.

### Definition of response

Imaging was reviewed according to RECIST version 1.1 at pretreatment, best response, DR (when applicable), and progression. Disease burden was divided into eight anatomic compartments: primary disease, cervical lymph nodes, other (noncervical) lymph nodes, lung, bone, liver, skin and soft tissue, and brain. Complete response (CR), PR, SD, and PD were assigned per RECIST version 1.1. DR was defined as objective response (CR or PR) in one anatomic compartment with simultaneous PD in another.

### Immunohistochemistry

We followed the methodology previously developed (16); slides were deparaffinized and rehydrated using graded concentrations of alcohol, followed by target retrieval solution (pH 9, #K8000 Dako/Agilent) at 98°C for 20 minutes. Slides were cooled to 85°C and washed in 1× wash buffer (#K8007, Dako). Staining was performed using a Dako Autostainer Link 48. Slides were incubated in Dual Endogenous Enzyme Block for 10 minutes and protein-free blocking solution (#X0909, Dako/Agilent) for 20 minutes, followed by the appropriate dilution of primary antibody and incubation for 60 minutes at room temperature: CD3 (Abcam #AB5690, RRID: AB_305055), CD4 (Agilent/Dako #M7310, RRID: AB_2728838), CD8 (Agilent/Dako #M7103, RRID: AB_2075537), interleukin enhancer–binding factor 3 (ILF3; Santa Cruz Biotechnology #SC377406, RRID: AB_3083533), programmed cell death ligand 1 (PD-L1; Cell Signaling Technology #13684, RRID: AB_2687655), phosphorylated signal transducer and activator of transcription 3 (pSTAT3; Abcam #AB76315, RRID: AB_1658549), and STAT3 (Cell Signaling Technology #4904, RRID: AB_331269). Staining was developed for 30 minutes with EnVision+ horseradish peroxidase–labeled polymer (#K4003 or K4001 Dako/Agilent, RRID: AB_2630375) for single staining and MACH 2 AP-Polymer (#RALP525L or #RALP521L Biocare Medical) for double staining. Slides were incubated in substrate-chromogen diaminobenzidine (DAB+) solution (#K3468 Dako/Agilent) for 10 minutes for single stains and in Vulcan Fast Red (VFR; #FR805S Biocare Medical) for 15 minutes for double stains. Slides were washed with 1× wash buffer after each reagent incubation and with distilled H_2_O after incubation with DAB and VFR and were counterstained with hematoxylin (#S3301 Dako/Agilent) for 5 minutes.

Prior to immunohistochemistry (IHC) analysis, all formalin-fixed, paraffin-embedded tissue blocks were reviewed by a trained pathologist to confirm the presence of HNSCC. Imaging and quantification were performed by investigators blinded to patient outcomes. To minimize interobserver variability, each endpoint was quantified entirely by a single researcher and spot-checked by a second researcher. For all endpoints, six representative images were acquired per slide at 10× (STAT3, pSTAT3, and ILF3), 20× (PD-L1), or 40× (CD3, CD4, and CD8) magnification. STAT3, pSTAT3, and ILF3 DAB staining intensity was quantified using ImageJ Fiji (RRID: SCR_002285) on a scale of 0 to 255. Tumor cell nests were manually selected while excluding infiltrating stromal cells prior to analysis, as previously described (17). PD-L1 expression was quantified using the CPS, calculated as the number of PD-L1–positive cells per 100 viable tumor cells, yielding scores ranging from 0 to 100 (18). Total immune cell counts (CD4^+^ and CD8^+^) were determined for each representative image, and compartment-specific analyses were performed using previously established criteria (19). Briefly, immune infiltration was assessed within the central tumor compartment while excluding the invasive margin (1 mm) because of variability in its presence and orientation among patient tissue blocks. Necrotic regions containing granulocytic infiltrates were also excluded from analysis. The proportion of each image occupied by tumor cell nests or stromal tissue was quantified to calculate the relative density of immune cell infiltration within each compartment.

### Proximity ligation assay

Proximity ligation assays (PLA) were performed as previously described (20) and quantified using BlobFinder V3.2 (RRID: SCR_015788; ref. 21). For each slide, five representative images were taken on a ZEISS Axio Observer Z1 inverted microscope (RRID: SCR_021351) using ZEISS Software Release 4.8 (ZEISS), with 15 z-stacked images per field of view. Antibodies used include PD-L1 (Cell Signaling Technology #13684, RRID: AB_2687655) at 1:200, ILF3 (Santa Cruz Biotechnology #SC377406, RRID: AB_3083533) at 1:100, and STAT3 (Abcam #AB68153, RRID: AB_2889877) at 1:400.

### Statistical analysis

Clinical endpoints were assessed using a two-sided *t* test, analysis of variance (ANOVA), or a *χ*^2^ test in GraphPad Prism version 9.0 (RRID: SCR_002798). IHC comparisons were made using a two-sided *t* test or one-way ANOVA. Survival outcomes were assessed using Kaplan–Meier survival analysis with log-rank comparisons. Subjects were censored at the time of last known follow-up. A multivariable logistic regression model was used to evaluate the association between the outcome variable DR and demographic and clinical variables. Clinical variables with zero DR events were not included in the model. *P* values no more than 0.05 were considered statistically significant.

## Results

### Treatment response types and associated patient characteristics

**Figure 1.**
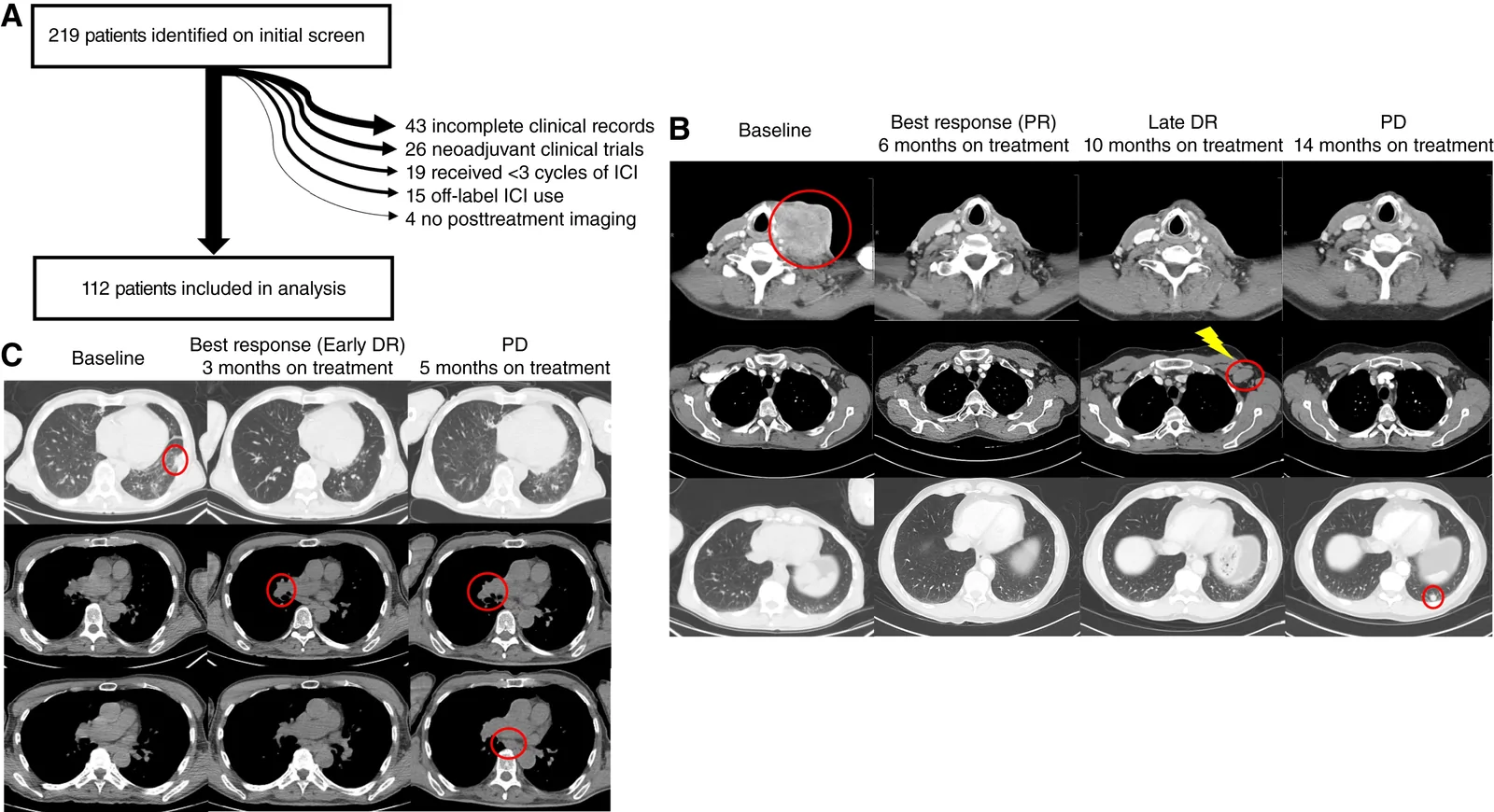
Patient selection process and DR examples. **A,** Initial screen identified patients with HNSCC treated with ICI. Patients were excluded if treated outside of the R/M setting, stopped ICI prior to receiving three cycles, or clinical records and/or imaging were incomplete. **B,** Radiology example of late DR. Patient had a large neck mass on baseline scans. Best response occurred at 6 months on treatment with substantial PR in the neck. Ten months after treatment start, the patient had a late DR with sustained PR in the neck but PD in the axillary lymph nodes which were treated locally with radiation. Overall PD occurred after 14 months on treatment with progression in the neck and diffusely through the lungs. **C,** Radiology example of early DR. Patient had a pleural-based mass on pretreatment scans. Best response and early DR occurred at 3 months on treatment with PR of the pleural-based lesion but PD in the right hilum. Patient continued on ICI alone and developed overall PD after 4 months on treatment with continued progression of hilar lymph nodes along with increased subcarinal and mediastinal lymphadenopathy.

One hundred and twelve patients with R/M HNSCC were included (Fig. 1A; Table 1). The patient population was overwhelmingly male (*n* = 88, 79%) with a median age of 64 years, and most had Eastern Cooperative Oncology Group performance status of 0 or 1 (*n* = 100, 89%). Oropharynx was the most common primary disease site (*n* = 47, 42%), followed by the oral cavity (*n* = 30, 27%), larynx (*n* = 18, 16%), nasal cavity and nasopharynx (*n* = 9, 8%), unknown (*n* = 5, 4%), and hypopharynx (*n* = 3, 3%). Forty-seven (42%) were positive for p16 by IHC which was performed in all but two oropharyngeal cases. ICI was most often given as the first line of treatment (*n* = 84, 75%) and as monotherapy (*n* = 83, 74%). Sites of R/M disease included lung metastases in 59 (53%), local recurrence in 54 (48%), cervical lymph nodes in 42 (38%), other lymph nodes in 45 (40%), bone in 16 (14%), liver in 6 (5%), skin and soft tissue in 7 (6%), and brain in 4 (4%) patients. Thirty-nine (35%) patients had disease in 1 organ system, 42 (37%) in 2 organs, 20 (18%) in 3 organs, and 11 (10%) had 4 or more systems affected (median organs involved 2; range, 1–6). Forty-three (38%) patients had recurrent disease in a previously irradiated field. The median follow-up was 12 months (range, 3–77 months).

**Table 1.** Clinical characteristics associated with treatment response.

Clinical characteristic	Response (SD, PR, or CR), *n* = 57 (51%)	No response (PD), *n* = 34 (30%)	DR, *n* = 21 (19%)	*P* value
Median age (years)	64	63	65	0.26
Male gender (%)	42 (74%)	30 (88%)	16 (76%)	0.25
Performance status	​	​	​	​
0	16 (28%)	12 (35%)	7 (33%)	0.41
1	34 (60%)	17 (50%)	14 (67%)	
2+	7 (12%)	5 (15%)	0	
Smoking status	​	​	​	​
Never smoker	19 (33%)	12 (35%)	9 (43%)	0.22
<10 pack-years	21 (37%)	6 (18%)	7 (33%)	
10+ pack-years	17 (30%)	16 (47%)	5 (24%)	
Primary disease site	​	​	​	​
Nasal cavity and nasopharynx	5 (9%)	4 (12%)	0	0.66
Oral cavity	13 (23%)	11 (32%)	6 (29%)	
Oropharynx	26 (45%)	12 (35%)	9 (43%)	
Hypopharynx	0	2 (6%)	1 (5%)	
Larynx	10 (18%)	4 (12%)	4 (19%)	
Unknown	3 (5%)	1 (3%)	1 (5%)	
p16 positive	25 (44%)	12 (35%)	10 (48%)	0.61
Line of treatment	​	​	​	​
1	44 (77%)	24 (71%)	16 (76%)	0.77
2+	13 (23%)	10 (29%)	5 (24%)	
Type of regimen	​	​	​	​
Monotherapy	38 (67%)	29 (85%)	16 (76%)	0.14
Combination therapy	19 (33%)	5 (15%)	5 (24%)	
Recurrence in prior RT field	26 (45%)	8 (24%)	10 (48%)	0.08
Metastatic disease site(s)	​	​	​	​
Local disease	26 (45%)	13 (38%)	15 (71%)	0.51
Cervical lymph nodes	18 (32%)	9 (26%)	15 (71%)	
Other lymph nodes	16 (28%)	19 (56%)	10 (48%)	
Lung	22 (39%)	23 (68%)	14 (67%)	
Bone	7 (12%)	6 (18%)	3 (14%)	
Liver	3 (5%)	2 (6%)	1 (5%)	
Skin and soft tissue	1 (2%)	3 (9%)	3 (14%)	
Brain	3 (5%)	1 (3%)	0	

NOTE: Patients were subdivided by radiographic response category. Patient characteristics were compared by ANOVA or *χ*^2^ test.

Clinical benefit from immunotherapy, including SD, PR, and CR, was observed in 68 (61%) patients. PD as the best response to treatment was seen in 44 (39%) patients. Twenty-one (19%) patients were found to have DR, defined as clinical benefit (CR or PR) by RECIST in one or more disease locations with concomitant growth meeting criteria for PD in one or more locations (Fig. 1B). At the time of DR, 8 (38%) patients were classified as having an overall clinical PR, 3 (14%) had SD, and 10 (48%) had PD. Twelve patients with DR (57%) continued immunotherapy beyond the DR timepoint—6 of these had no escalation of therapy, 4 had RT to oligometastatic sites, 1 had surgical resection, and 1 added chemotherapy to the existing ICI.

### DR association and differential outcomes

Primary disease site, p16 status, prior RT, and ICI monotherapy were not associated with response type (Table 2). The most significant predictors of DR in multivariable logistic regression modeling were sites of metastatic disease: DR was more likely to occur in patients who had metastases to cervical lymph nodes [odds ratio (OR), 16.79; 95% confidence interval (CI), 2.96–157.10; *P* = 0.004], lung (OR, 4.99; 95% CI, 1.16–26.49; *P* = 0.041), or skin/soft tissue (OR, 23.93; 95% CI, 1.91–470.68; *P* = 0.020). We also observed distinct differences in clinical course based on when the DR occurred. We identified two patient subgroups: DR at the time of best response (early DR, *n* = 11) and uniform initial response followed by DR at progression (late DR, *n* = 10; Supplementary Fig. S1; Supplementary Table S1). No patient with early DR converted to overall response on subsequent imaging: 8 of 11 (73%) progressed within 3 months, and the remaining 3 progressed within 6 months.

**Table 2.** Independent effects of clinical variables on the probability of DR.

Variable	OR	95% CI	*P* value
Age	1	0.94–1.07	0.998
Gender	0.82	0.14–4.50	0.823
Active or heavy tobacco use	0.32	0.06–1.59	0.175
P16 positivity	6.52	0.54–82.12	0.136
ICI monotherapy	0.84	0.18–3.61	0.817
Recurrence in RT field	1.95	0.46–8.95	0.372
Local disease recurrence	3.38	0.78–16.90	0.115
Cervical lymph node metastases	9.39	2.26–51.25	0.004
Lung metastases	4.52	1.18–19.87	0.034
Skin/soft-tissue metastases	19.52	1.91–259.42	0.015

NOTE: A multivariate logistic regression model was used to evaluate the association between the outcome variable DR and demographic and clinical variables. The effects of some major variables are summarized in the table above.

### Organ-specific response rates and survival outcomes

**Figure 2.**
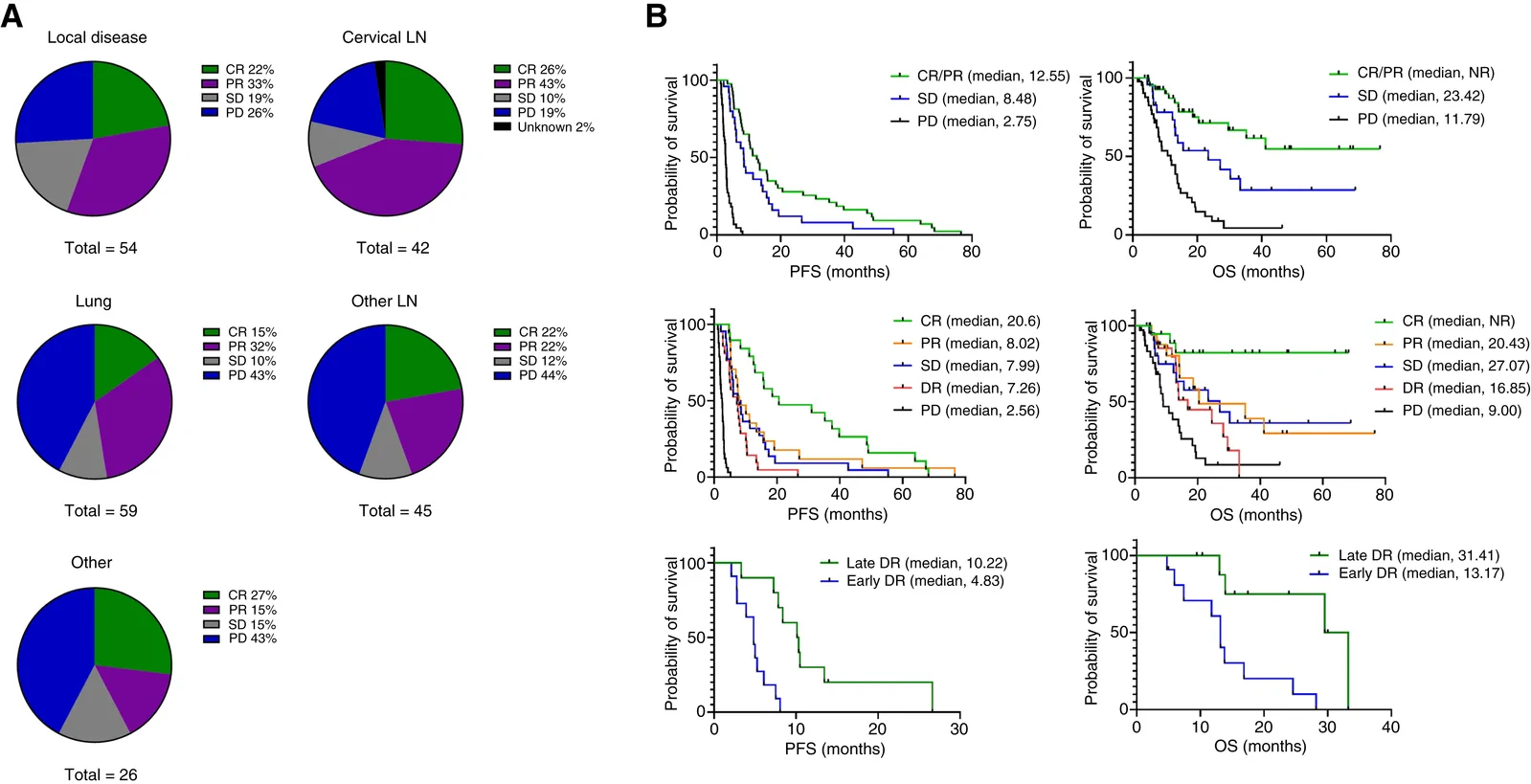
Organ-specific treatment response rates and impact of overall treatment response on survival outcomes. **A,** Organ-specific treatment responses for local disease (*n* = 54), cervical lymphadenopathy (*n* = 42), lung metastases (*n* = 59), other (noncervical) lymph nodes (LN; *n* = 45), and other sites of disease (bone, liver, skin and soft tissue, and/or brain, *n* = 26). **B,** Kaplan–Meier estimates of PFS (left) and OS (right) in months. Top, RECIST response classifications. Middle, subdivided response categories demonstrated that DR was similar to SD and PR with respect to clinical outcomes. Bottom, late DR had more favorable impact on survival than early DR. There were 86 PFS events and 61 OS events in the total cohort, and 19 PFS events and 14 OS events in the DR subgroup. Tick marks indicate censored data.

To better understand ICI response profiles, we calculated organ-specific treatment response rates for different sites of disease across the entire patient population (Fig. 2A). The objective response rates (ORR) were 55% for primary site recurrences, 69% for cervical lymphadenopathy, 44% for other lymphadenopathy, 47% for lung metastases, and 42% for metastases to other sites including the bone, liver, brain, skin, and soft tissue. The rate of PD was highest for disease sites outside the head and neck. In patients with DR, there was a pattern of higher ORR in the cervical lymph nodes (73%) and lungs (67%) but decreased ORR for local disease recurrence (40%), noncervical (typically thoracic) lymphadenopathy (31%), and other sites including the bone, liver, skin, and soft tissue (33%; Supplementary Table S2). Twelve (57%) had progression in a single organ, whereas 9 had progression in 2 or more organs at the time of DR.

We then evaluated the impact of overall and organ-specific treatment response type on progression-free survival (PFS) and OS. As demonstrated in Fig. 2B, best response of CR was associated with superior PFS (median, 20.6 months; *P* < 0.0001) and OS [median, not reached (NR); *P* < 0.0001] when compared with all other groups. Interestingly, patients with PR, SD, and DR did not differ significantly from each other in their survival outcomes. Patients with DR had statistically better PFS (median, 7.3 vs. 2.6 months; *P* < 0.0001) and OS (median, 16.9 vs. 9 months; *P* = 0.041) versus patients with uniform PD. Within the DR subgroup, patients with late DR had significantly improved PFS (median, 10.22 vs. 4.83 months; *P* = 0.005) and OS (median, 31.41 vs. 13.17 months; *P* = 0.008) versus patients with early DR. Of the various disease sites, organ-specific response in the lungs was the most predictive of good overall outcome with *P* < 0.0001 for both PFS and OS (Supplementary Fig. S2).

### ICI response is associated with increased infiltration of CD8^+^ T cells

**Figure 3.**
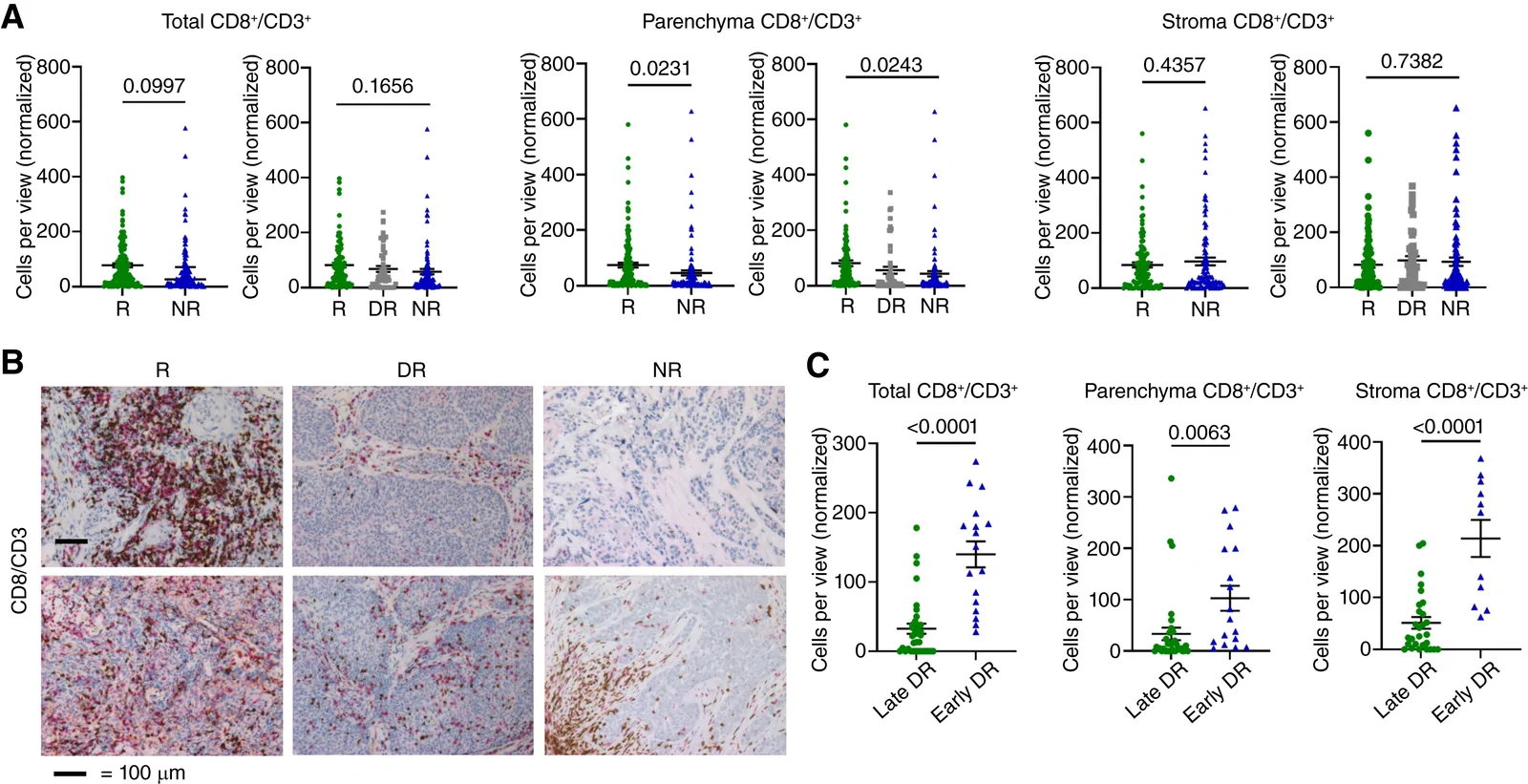
TILs are associated with treatment response patterns. **A,** Quantification and (**B**) IHC images of CD8^+^ T cells showed increased infiltration in the parenchyma of responders (R) vs. nonresponders (NR) or dissociated responders. No differences were observed in the stroma. Brown, CD8; red, CD3. **C,** Early DR cases demonstrated higher CD8^+^ T-cell invasion compared with late DR cases in both the parenchyma and stroma. Five representative images per case were quantified; lines represent mean, and errors bars represent standard error of the mean. DR, dissociated response.

We identified 45 patients who had tissue available for analysis, including 18 (40%) responders (CR, PR, or SD), 9 (20%) patients with DR, and 18 (40%) nonresponders. Twenty-five (56%) samples were biopsies or surgical resections from the time of initial diagnosis and management; the majority of these samples (18) were from the primary mucosal disease site with the remainder coming from lymph nodes (3) or soft-tissue/skin metastases (4). The other 20 (44%) were obtained within 3 months of starting ICI including 7 from locally recurrent disease, 3 from lymph nodes, 2 from soft-tissue or skin metastases, and 8 from distant organ metastases. Increased tumor-infiltrating lymphocytes (TIL), defined as CD8^+^/CD3^+^, was observed in the tumor parenchyma in samples from responders compared with nonresponders (Fig. 3A and B). Stroma infiltration was not significantly different. DR patient tissue was overall more similar to nonresponding than responding tumors with respect to CD8^+^ T-cell infiltration. However, total T-cell infiltration was higher in patients with early DR than late DR including substantial differences in both the parenchyma and stroma (Fig. 3C). In three patients with paired samples available for comparison, CD8^+^ T-cell invasion declined upon recurrence (Supplementary Fig. S3A). This phenomenon was seen in both ICI responders and nonresponders.

**Figure 4.**
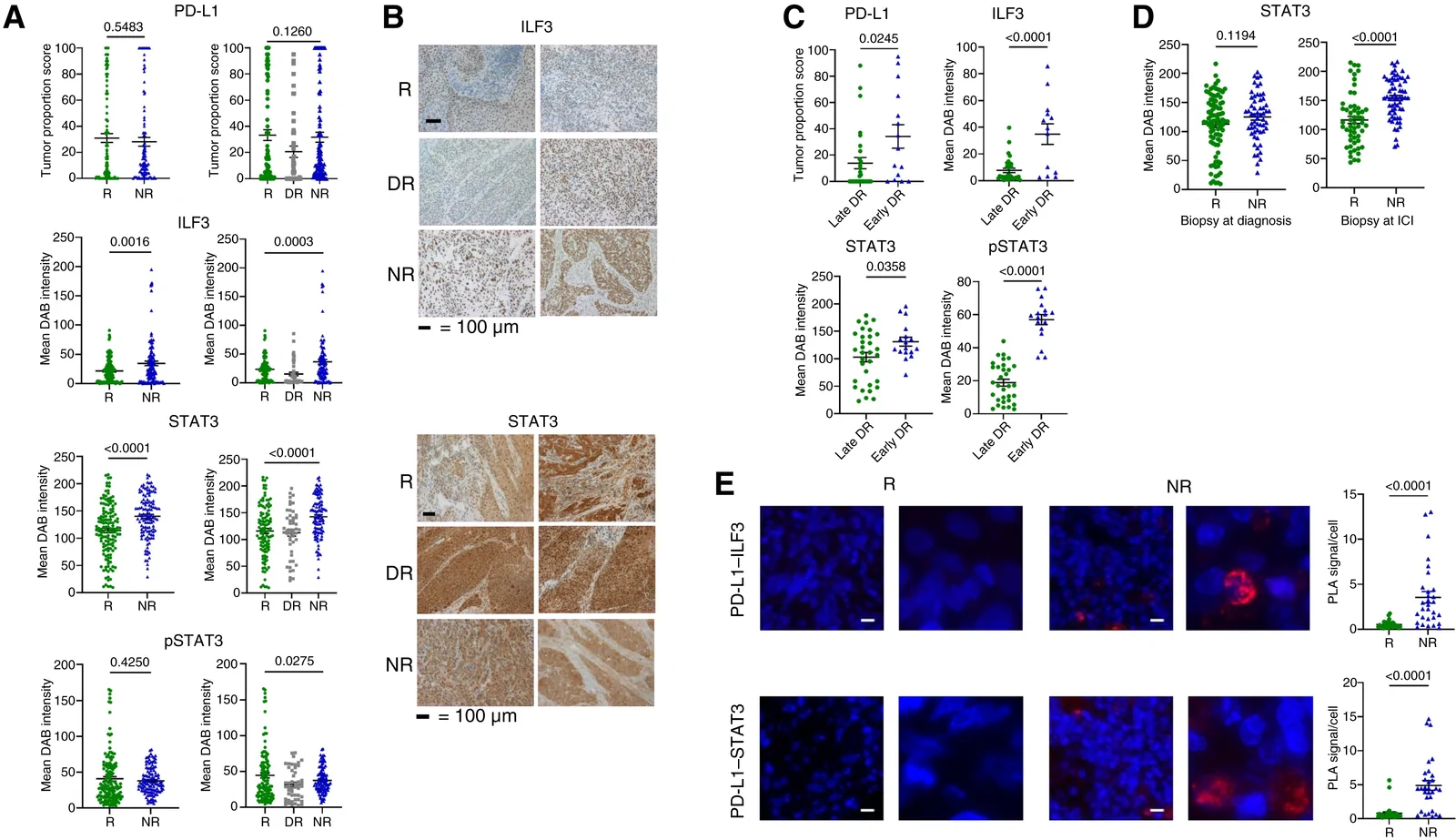
Differential activity of PD-L1 interactome signaling based on tumor response. **A,** Quantification of PD-L1, ILF3, STAT3, and pSTAT3 showed increased ILF3 and STAT3 expression in nonresponding tumors. NR, nonresponders; R, responders. **B,** Representative IHC images of ILF3 and STAT3 staining by response type. **C,** All components of the PD-L1 interactome were increased in early compared with late DR cases. Five representative images per case were quantified; lines represent mean, and errors bars represent standard error of the mean. **D,** Top, differences in STAT3 expression by treatment response were specific to tissue samples collected just prior to ICI initiation. **E,** Multicolor PLA representative images (left) and quantification (right) revealed significantly higher PD-L1 intracellular signaling activity in nonresponding vs. responding cases as demonstrated by increased interaction between PD-L1–ILF3 and PD-L1–STAT3. Five cases per group with quantification from five representative images. Scale bars, 10 μm (left); magnified PLA is 4× original size.

We next used IHC to evaluate the intrinsic PD-L1 interactome signaling cascade in responding and nonresponding patients. We have previously shown a role for PD-L1 in inducing coordinated and simultaneous T-cell anergy and increased cancer cell aggressiveness with patient-derived HNSCC models, which occurs through binding of the PD-L1 intracellular domain to ILF3 causing increased ILF3 nuclear translocation and subsequent activation of STAT3 (16). PD-L1 tumor score and pSTAT3 were similar across response types (Fig. 4A). However, and consistent with our preclinical data, ILF3 and STAT3 were both significantly increased in nonresponders (Fig. 4A and B). Tissue interactome signaling in DR patients was more similar to that in responders than to that in nonresponders (Fig. 4A). Within this group, PD-L1, ILF3, STAT3, and pSTAT3 were uniformly higher in early DR compared with late DR, suggesting higher progrowth signaling consistent with poorer outcomes (Fig. 4C). The difference in STAT3 expression between response types manifested at the time of immunotherapy initiation; there was no relationship between ICI response and STAT3 expression in earlier tissue samples (Fig. 4D). Interestingly, STAT3 increased over time/with recurrence in both responders and nonresponders in the limited set of patients who had serial tissue available for comparison (Supplementary Fig. S3B).

Physical interaction between PD-L1 and ILF3, as well as PD-L1 and STAT3, was confirmed using a PLA, with substantially increased signal present in the nonresponding group (Fig. 4E). These results validate in patient tissue both the mechanistic basis and the hypothesized signaling pattern of the PD-L1 interactome in HNSCC.

## Discussion

We have characterized the phenomenon of DR in patients with R/M HNSCC treated with ICI, finding a prevalence of 19%. This is significant from a practice standpoint because these patients were evenly distributed between those deemed potentially deriving benefit and those deemed to be progressing overall, indicating that this is a distinct subgroup in a clinical “gray” area. Both clinical benefit and DR incidence may be overestimated in our cohort given strict patient selection criteria: Patients had to have at least two imaging studies spanning at least three ICI cycles for inclusion, likely selecting against those with rapid progression and/or deteriorating performance status.

Interestingly, patients with DR had survival outcomes more comparable with SD than PD, suggesting that the DR category of response denotes clinical benefit. These findings support the continued use of ICI after late DR when local therapies can be used to address limited sites of progression, especially in those patients who initially have a CR or PR. Conversely, change or escalation of therapy should be considered for patients with early DR as we did not identify any instance in which early DR converted to a uniform response. Early DR may be explained by organ-specific differences in the TME leading to intrinsic resistance, whereas late DR could be akin to the clonal selection resulting in acquired resistance seen in other solid tumors, most notably lung cancer. The dearth of oncogenic driving mutations in HNSCC makes comparative genetic exploration of such cases a valuable next step.

When assessing clinical factors that may predict ICI response, we found the pattern of organ involvement to be a significant variable. Although cervical lymphadenopathy was a strong positive predictor of DR, we were surprised to find no relationship between prior RT and response pattern. This is similar to the findings of Vaflard and colleagues (22), who reported on DR patterns in 100 patients including 43 with head and neck cancers. The lack of association may be due to small sample size or to the complex interplay among tumor, microenvironment, and conventional therapies. The impact of radiation on the local TME is not fully understood but is generally thought to be immunosuppressive (23), which is supported by our finding that 7 of 10 (70%) patients with DR and in-field recurrence experienced PD at that site. These findings also suggest that prior reports of lower ICI response in patients with limited locoregional recurrence may reflect an increase in DR rather than uniform PD, a nuance not well captured in trials reporting strictly per RECIST.

Irrespective of DR, we found noteworthy patterns of response to ICI in R/M HNSCC. The decreased response rates (possibly secondary to “colder” TME) in in-field relapses carry an important clinical implication, as in day-to-day clinical practice the choice is between using cytotoxic agents along with re-RT as salvage versus initiating ICI in cases of limited locoregional recurrence. An emerging regimen combining stereotactic body RT with ICI has also demonstrated efficacy in this setting and may yield further insights into the optimal combination and sequencing of these modalities (24). Also, the different prognostic impact of a neck node versus mediastinal node versus lung metastasis response is both hypothesis generating and seemingly shared across multiple solid tumor types. This should foster enhanced scrutiny to the respective TMEs while directing possible precision therapy combinations to be deployed based on the pattern of disease.

**Figure 5.**
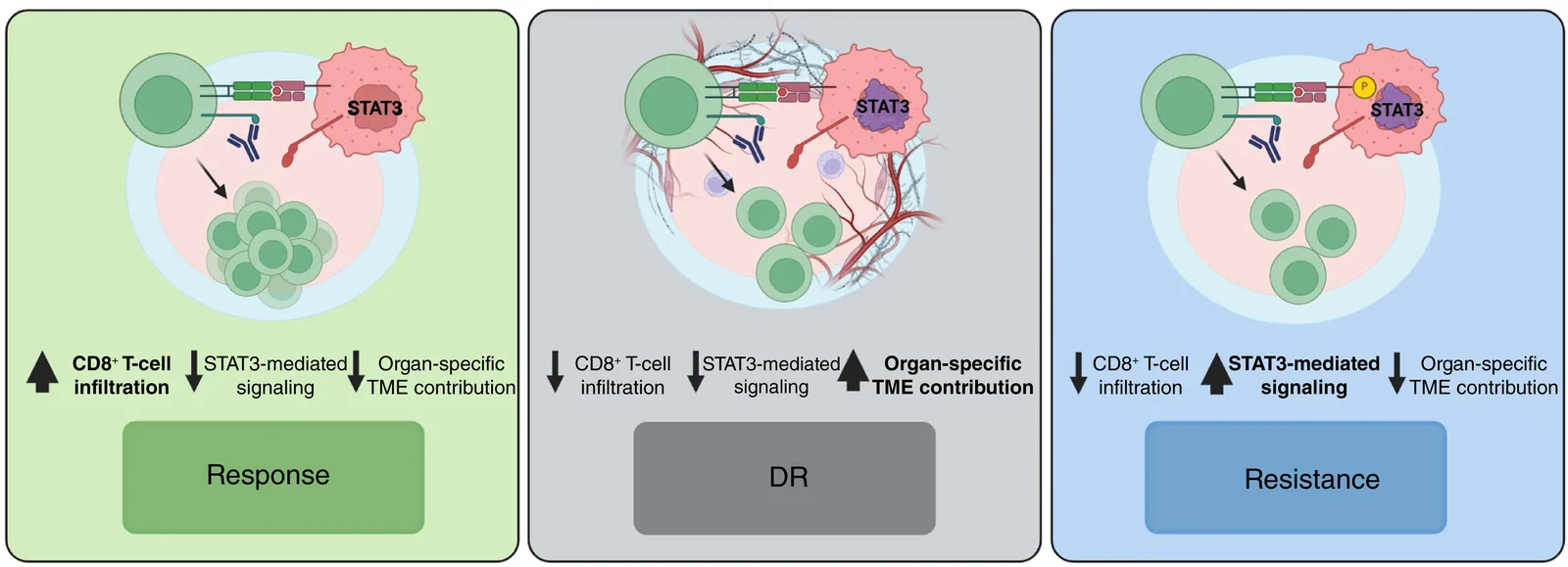
Response patterns to immune checkpoint inhibition in HNSCC. We hypothesize that patients treated with immune checkpoint inhibitors (ICI) can be categorized into three response patterns: uniform response, characterized by high CD8^+^ T-cell infiltration and low STAT3-mediated signaling with limited organ-specific variability; DR, typified by low CD8^+^ T-cell infiltration and STAT3-mediated signaling with marked organ-specific variability; and uniform resistance, identified by low CD8^+^ T-cell infiltration and high STAT3-mediated signaling with limited organ-specific variability.

Along that theme, we confirmed the importance of both TILs and PD-L1–driven cancer signaling in mediating ICI efficacy. Responders had increased CD8^+^ T-cell infiltration and reduced signaling along the PD-L1–STAT3 axis compared with nonresponders. Patients with DR were found to have an intermediate immune phenotype with lower levels of both PD-L1–STAT3 signaling and CD8^+^ T cells, suggesting a greater role for organ-specific TME factors or a more immune-cold phenotype (Fig. 5). In both uniform nonresponders and patients with DR, a developing hypothesis is that ICI response could be optimized by the addition of therapies targeting STAT3 and/or other mediators of PD-L1 intracellular signaling–mediated treatment resistance. Whether this is best achieved with up-front combinations or by escalation upon resistance/progression is a fascinating question; the fact that pretreatment STAT3 was higher in nonresponders indicates a possible baseline trait as opposed to an acquired response, favoring an up-front combinatorial strategy. These questions frequently arise in the day-to-day management of R/M HNSCC and have become acutely relevant with the approval of ICI in the management of curable HNSCC suitable for surgical resection (25, 26). Dealing with DR and hyper-PD in this setting represents new clinical conundrums for HNSCC clinicians.

In summary, here we describe the phenomenon of DR to ICI in R/M HNSCC. This includes early DR, which seems to be a precursor to uniform disease progression, and late DR, which seems associated with improved survival outcomes. Organ-specific discrepancies in ICI response likely reflect differences in the TME linked to the PD-L1 interactome and T-cell infiltration. Our analysis is limited by the modest number and heterogeneous characteristics of the patients and tumors described, including primary disease site, pattern of metastatic organ involvement, and sequencing of treatment modalities. These constraints replicate clinical practice, and findings are intended to be thought-provoking for physicians treating patients and designing clinical trials for R/M HNSCC. Additional investigation is needed to elucidate the relative contributions of tumor and immune signaling to help guide combination immunotherapy approaches to improve the efficacy of treatment.

## Supplementary Material

Supplemental Figure 1 Supplemental Figure 1. Timing of treatment responses in patients with DR. Timing of best response (red), dissociated response (DR, orange), and overall progression of disease (PD, blue) in months from the time of first immunotherapy cycle in patients with dissociated response. Those patients where best response and dissociated response occurred at the same time were classified as early DR (grouped at the top), and those patients where dissociated response occurred at least 1 month after best response were classified as late DR (grouped at the bottom).

Supplemental Figure 2 Supplemental Figure 2. Survival outcomes based on lung-specific treatment response. Kaplan-Meier estimates of progression free survival (PFS, left) and overall survival (OS, right) in months based on organ-specific treatment response of lung metastases. Tick marks indicate censored data. Abbreviations: complete response (CR), not reached (NR), partial response (PR), stable disease (SD), and progressive disease (PD).

Supplemental Figure 3 Supplemental Figure 3. Tissue characteristics in patients with paired samples available from diagnostic biopsy/surgery, salvage biopsy/surgery, and time of ICI initiation. A. CD8+ T cell infiltration decreased over time in both responders and non-responders with serial tissue available for comparison. Shown are the averages of 5 representative images at each timepoint. B. STAT3 increased over time in both ICI responders and non-responders with serial biopsies available for comparison (bottom row).

Supplemental Table 1 Supplemental Table 1. Clinical characteristics associated with timing of DR.

Supplemental Table 2 Supplemental Table 2. Response by disease site in patients with DR.

## Data Availability

The data generated in this study are not publicly available owing to patient privacy requirements but are available upon reasonable request to the corresponding author.
