# Supplementary material for: Dissociated Response to Immune Checkpoint Inhibition in Head and Neck Squamous Cell Carcinoma Is Common and Exhibits Distinct Clinical Outcomes
Source: Cancer Res Commun. 2026 Oct 8;6(10):2350–9. doi: 10.1158/2767-9764.CRC-26-0363 (PMC13647153; doi:10.1158/2767-9764.CRC-26-0363)
Supplement: Supplementary file 1 [file crc-26-0363_supplemental_table_1_suppst1.docx]

| **Clinical Characteristics** | | **Early DR**  **n=11** | **Late DR**  **n=10** | **P-value** |
| --- | --- | --- | --- | --- |
| **Median Age (yrs)** | | 65 | 67 | 0.45 |
| **Male Gender (%)** | | 8 (73%) | 8 (80%) | 0.99 |
| **Performance Status** | **0** | 4 (36%) | 3 (30%) | 0.77 |
|  | **1** | 7 (64%) | 7 (70%) |  |
| **Smoking Status** | **Never Smoker** | 5 (45%) | 4 (40%) | 0.82 |
|  | **<10 Pack Years** | 3 (21%) | 4 (40%) |  |
|  | **10+ Pack Years** | 3 (27%) | 2 (20%) |  |
| **Primary Disease Site** | **Oral Cavity** | 3 (27%) | 3 (30%) | 0.07 |
|  | **Oropharynx** | 7 (64%) | 2 (20%) |  |
|  | **Other** | 1 (9%) | 5 (50%) |  |
| **p16 Positive** | | 7 (64%) | 3 (30%) | 0.12 |
| **Line of Treatment** | **1** | 7 (64%) | 9 (90%) | 0.15 |
|  | **2+** | 4 (36%) | 1 (10%) |  |
| **Type of Regimen** | **Monotherapy** | 10 (91%) | 6 (60%) | 0.10 |
|  | **Combination Therapy** | 1 (9%) | 4 (40%) |  |
| **Recurrence in Prior RT Field** | | 4 (36%) | 6 (60%) | 0.24 |
| **Metastatic Disease Site(s)** | **Local Disease** | 6 (55%) | 9 (90%) | 0.68 |
|  | **Cervical Lymph Nodes** | 8 (57%) | 7 (70%) |  |
|  | **Other Lymph Nodes** | 7 (64%) | 3 (30%) |  |
|  | **Lung** | 8 (73%) | 6 (60%) |  |
|  | **Other** | 4 (36%) | 3 (30%) |  |

**Supplemental Table 1. Clinical characteristics associated with timing of DR..** Patients were subdivided by timing of DR: DR at the time of best response (early DR, n=11), and uniform initial response followed by DR at progression (late DR, n=10). Patient characteristics were compared by ANOVA or chi-square test. Abbreviations: dissociated response (DR), radiation therapy (RT).
