# Supplementary material for: Dissociated Response to Immune Checkpoint Inhibition in Head and Neck Squamous Cell Carcinoma Is Common and Exhibits Distinct Clinical Outcomes
Source: Cancer Res Commun. 2026 Oct 8;6(10):2350–9. doi: 10.1158/2767-9764.CRC-26-0363 (PMC13647153; doi:10.1158/2767-9764.CRC-26-0363)
Supplement: Supplementary file 2 [file crc-26-0363_supplemental_table_2_suppst2.docx]

| **Primary Site** | **Local Disease** | **Cervical LN** | **Other LN** | **Lung** | **Other** |
| --- | --- | --- | --- | --- | --- |
| Larynx | CR | PD |  |  |  |
| Oral Cavity | CR | PR | PD | PR |  |
| Unknown | CR |  | PD | PR |  |
| Oropharynx | PR | PR | PD | PD |  |
| Larynx | PR | PR | PD | PD |  |
| Oropharynx | SD | PR |  |  | PD |
| Oral Cavity | PD | CR |  |  |  |
| Oropharynx | PD | CR | CR | PD |  |
| Oral Cavity | PD | PR |  |  |  |
| Oropharynx | PD | PR | CR |  |  |
| Hypopharynx | PD | PD | CR |  | CR |
| Oropharynx | PD | PD |  | CR |  |
| Oral Cavity | PD |  | PD | PR |  |
| Larynx | PD |  | PD | PR | PD |
| Oral Cavity | PD |  |  | PR | CR |
| Oral Cavity |  | CR |  | PD |  |
| Oropharynx |  | PR | PD | PD |  |
| Larynx |  | PR |  | PR | PD |
| Oropharynx |  | PD | PD | PR |  |
| Oropharynx |  |  | PR | PR | PD |
| Oropharynx |  |  | PD | PR |  |

**Supplemental Table 2. Response by disease site in patients with DR.** Abbreviations: dossciated response (DR), lymph node (LN), complete response (CR), progressive disease (PD), partial response (PR), stable response (SD).
